# Rootstock genotype drives a metabolic trade-off between phenylpropanoids and terpenoids in *Camellia sinensis*

**DOI:** 10.3389/fpls.2026.1781298

**Published:** 2026-04-16

**Authors:** Cheng Ma, Yanchun Zheng, Yaxin Deng, Peng Zheng, Hongbo Zhao, Shaoqun Liu, Binmei Sun

**Affiliations:** 1 College of Horticulture, South China Agricultural University, Guangzhou, Guangdong, China; 2 Guangdong Provincial Engineering Technology Research Center for Southern Specialty Tea, Guangzhou, Guangdong, China

**Keywords:** *Camellia sinensis*, flavor, grafting, metabolic trade-off, phenylpropanoids, terpenoids, transcriptome

## Abstract

In perennial crops, a metabolic trade-off between defense-related and aroma-related compounds fundamentally shapes quality, yet its regulation remains elusive. Grafting is a key horticultural technique that can alter this balance in tea plants (*Camellia sinensis*), but the underlying molecular mechanisms are unclear. To decipher this rootstock-driven trade-off, we employed an integrated multi-omics approach in *Camellia sinensis* cv. ‘Yashixiang Dancong’ and *Camellia sinensis* cv. ‘Lingtou Dancong’. Scions of the high-aroma cultivar ‘Yashixiang’ were grafted onto the vigorous ‘Lingtou’ rootstocks (hetero-grafting) and self-grafted (homo-grafting). We observed a pronounced metabolic trade-off: hetero-grafting significantly reduced phenylpropanoids (catechins) and caffeine (bitter-tasting compounds) while markedly enriching volatile terpenoids and fatty acid derivatives (aroma compounds). Transcriptome analysis revealed that this shift was orchestrated by a systematic transcriptional reprogramming mediating the trade-off: key gateway genes in the phenylpropanoid and caffeine pathways (*CsPAL*, *Cs4CL*, *CsTCS*) were downregulated, creating a bottleneck that limited flux toward non-volatile metabolites. Concurrently, genes encoding rate-limiting enzymes in the terpenoid backbone pathways (*CsHMGR*, *CsDXS*) were upregulated, enhancing precursor supply for volatile synthesis. This demonstrates that the rootstock genotype directs a precise metabolic trade-off in the scion, prioritizing aroma (terpenoids) production over defense (phenylpropanoids) accumulation. Our findings elucidate the molecular basis of grafting-modulated flavor quality and provide a framework for harnessing rootstock-scion interactions to fine-tune metabolic trade-offs in perennial crops.

## 1 Introduction

The tea plant (*Camellia sinensis*) is an evergreen perennial woody plant (Chen et al., 2007), and the beverage made from its fresh leaves is one of the most popular beverages in the world. The flavor quality of tea and its health benefits are primarily attributed to the abundant secondary metabolites in the leaves, such as catechins (Khan and Mukhtar, 2019), caffeine (Manolis et al., 2022), amino acids (Tang et al., 2019), and volatile aromatic compounds (Fu et al., 2024). The types and concentration of these compounds not only determine the taste, aroma, and color of tea, but also constitute the core of its economic value.

Grafting is a traditional horticultural technique widely used in tea plant cultivation (Tuwei et al., 2008; Ning et al., 2014). Its main purposes are to enhance the stress resistance of tea plants (e.g., drought and poor soil resistance) (Karunakaran and Ilango, 2019; Cheng et al., 2025), replace seed propagation to maintain the excellent characteristics of varieties (Ning et al., 2014), and improve propagation efficiency and production rates (Karunakaran and Ilango, 2019). However, grafting—as a technique that artificially connects the vascular systems of rootstock and scion—inevitably alters material transport and information exchange, potentially profoundly influencing the metabolic profiles of the scion portion (Kyriacou et al., 2017; Feng et al., 2024). Previous studies have shown that grafting can significantly alter the accumulation of metabolites in the scion. For example, cucumbers grafted onto different rootstocks exhibit significant changes in primary metabolites such as sugars, organic acids, amino acids, and alcohols (Miao et al., 2019); rootstocks can also influence the composition of sugars, acids, and volatile components in tomato flesh (Zhou et al., 2022). Additionally, grafting can regulate the expression of various stress response genes (Wang et al., 2017; Sharma and Zheng, 2019). Nevertheless, in tea plants, although biochemical changes have been observed after grafting, the transcriptional and metabolic regulatory networks associated with flavor-related components remain unclear. Critically, previous studies have often focused on general compositional changes, and the central question of a rootstock-mediated “metabolic trade-off”—wherein the synthesis of defense-related compounds (e.g., catechins) is potentially suppressed to favor volatile aroma production—remains mechanistically obscure. This knowledge gap is particularly salient in the context of ‘Dancong’ cultivars, a unique genetic group prized for its intense floral aroma (Chen et al., 2022). Here, the potential metabolic trade-off between astringency (primarily from catechins) and floral aroma (largely from terpenoids) is of paramount importance for quality, yet how rootstocks precisely modulate this balance remains unexplored. Specifically, the cultivar ‘Yashixiang’ (literally ‘Duck Shit Fragrance’) is renowned for its intense, gardenia-like floral aroma and is considered a benchmark for premium quality, though it requires specific cultivation conditions. In contrast, ‘Lingtou’ (also known as ‘Baiye’) is distinguished by its vigorous root system and broad environmental adaptability, attributes that make it a widely used rootstock. Therefore, the combination of ‘Yashixiang’ scions with ‘Lingtou’ rootstocks constitutes an ideal experimental system to directly test the hypothesis that a vigorous rootstock imposes a systematic metabolic trade-off, repressing defense-related phenylpropanoids in favor of aroma-related volatiles in the scion.

To address this, our study employed an integrated multi-omics approach to decipher the molecular basis of the rootstock-driven metabolic trade-off between phenylpropanoids and terpenoids in grafted tea plants. Using a specific high-aroma tea plant system, we tested the hypothesis that rootstocks act as a metabolic filter that reallocates biosynthetic resources. This study provides novel insights into the transcriptional and metabolic mechanisms underlying this trade-off, offering a theoretical foundation for harnessing grafting to precisely modulate secondary metabolism for quality improvement.

## 2 Materials and methods

### 2.1 Plant materials and growth conditions

The experimental materials in this study were all from ‘Dancong’ tea plants of Sanjin Tea Industry in Raoping County, Chaozhou City, Guangdong Province, China. The tea plants utilized for grafting were identified as ‘Lingtou Dancong’ (hereafter LD) and ‘Yashixiang Dancong’ (hereafter YD), both of which were five years old (**Supplementary Figure 1**). The surgical procedure employed was low-position cleft grafting (**Supplementary Figure 2**), performed in December 2022. The specific grafting scheme involved graftingYD scions ontoLD rootstock tea plants (YL, hetero-grafting) and grafting YD scions onto YD rootstock tea plants (YY, homo-grafting) (**Supplementary Figure 3**), with consistent management practices applied to the grafted tea plants.

After one year of grafted seedling management, sampling was conducted in December 2023. The one-year time point was selected because it represents a critical phase in graft union development when vascular connections are fully established and rootstock-scion signaling has stabilized, yet before confounding effects from long-term environmental adaptation may obscure grafting-specific metabolic responses (Goldschmidt, 2014; Feng et al., 2024). Sampling was performed during the winter dormant period to minimize interference from active growth and seasonal fluctuations in secondary metabolism. The “one bud and three leaves” standard was adopted as this represents the typical harvesting standard for mature tea leaves where secondary metabolites, particularly catechins and volatile compounds, are fully accumulated and compositionally stable (Chen et al., 2022). All samples were collected between 9–11 a.m. on a clear day to minimize diurnal variation. Environmental parameters at the time of sampling were recorded: photosynthetically active radiation (PAR) = 850-950 μmol·m^−2^·s^−1^, air temperature = 18-22 °C, soil pH = 5.2 ± 0.3, soil organic matter content = 2.8 ± 0.4%. These environmental conditions were consistent across all blocks, ensuring that observed metabolic differences reflect genotypic effects rather than environmental variation. The grafting success rate was >70% in all combinations, and all grafted plants exhibited normal growth with fully developed vascular union at the time of sampling. The tea samples were promptly dried, preserved with dry ice and transferred to a -80 °C refrigerator upon arrival at the laboratory for storage and future use.

### 2.2 Determination of the taste component contents in tea leaves

The fresh tea leaves were ground to powder under liquid nitrogen conditions and vacuum freeze-dried by vacuum freeze dryer (LGJ-18S, Songyuan Freeze Dryer). The freeze-dried tea powder was used for metabolites detection. The determination of catechins and caffeine content was performed according to the method described by (Chen et al., 2022).

The contents of C, EC, EGC, ECG, and EGCG were analyzed by Waters Alliance Series HPLC system (Waters Technologies, Milford, MA, USA) (Li et al., 2022). Briefly, 0.20 g tea powder in 8 mL 70% methanol was sonicated (Xinzhi Biological Technology Co., Ltd., Ningbo, China) for 30 min and centrifuged at 13,000 rpm for 10 min. One ml of the supernatant was filtered through a 0.22 µm Millipore membrane and then injected into an XSelect HSS C18 SB column (4.6 × 250 mm, 5 mm, Waters Technologies, Milford, MA, USA) maintained at 25 ± 5 °C. A gradient elution program, employing mobile phases A (0.1% aqueous formic acid) and B (100% acetonitrile), was executed in accordance with the following protocol: 0–5 min, 8–25% B; 14–30 min, 8% B. Catechins were detected at 280 nm, and the flow rate was 1 mL/min. The content of C, EC, EGC, ECG, and EGCG were identified through a comparison of their retention time and absorption spectrum with those of an authentic standard. All analyses were performed with three biological replicates, each measured with three technical replicates. The mean values were calculated from the biological replicates.

The caffeine content was analyzed using an HPLC (Alliance E2695, Waters) coupled to a 2489 UV/Vis detector (Waters Technologies, Milford, MA, USA) (Mei et al., 2022). 30 mL of 1.5% magnesium oxide in ultrapure water (w/v) was added to 0.10 g tea powder in a 50 mL centrifuge tube and extracted for 30 min at 100 °C. After centrifugation at 13,000× g for 10 min, 1 mL of the supernatant was filtered through a 0.22 μm nylon membrane (Jinteng Experimental Equipment Co., Ltd., Tianjin, China). A volume of 10 μL of the filtrate was injected into an XSelect HSS C18 SB column (4.6 × 250 mm, 5 mm, Waters Technologies, Milford, MA, USA) maintained at a temperature of 35 ± 1 °C and at a flow rate of 0.9 mL/min. The elution of caffeine was conducted under isocratic conditions. The mixture consisted of 30% A (100% methanol) and 70% B (ultrapure water), and detection occurred at 280 nm. The caffeine content was identified through a comparison of its retention time and absorption spectrum with the standard. All analyses were performed with three biological replicates, each measured with three technical replicates.

The quantification of free amino acids was conducted in accordance with the protocol stipulated in the Chinese National Standard GBT8314-2013, with minor modifications. Briefly, 1.5 g of tea powder was extracted with 225 mL of ultrapure water at 100 °C for 45 min. This test solution was composed of an extract, washing solution, and ultrapure water, mixed to a total volume of 250 mL. To 1 mL of the solution, 0.5 mL of pH 8.0 buffer (22.71 mg/mL disodium hydrogen phosphate dodecahydrate and 0.46 mg/mL potassium dihydrogen phosphate in ultrapure water) and 0.5 mL of 2% ninhydrin (diluted in ultrapure water with 0.8 mg/mL stannous chloride) were added. The mixture was incubated in a boiling water bath for 15 min, and ultrapure water was added to 25 mL after cooling. Absorbance was measured at 570 nm using a UV spectrophotometer (Shimadzu Instruments, Suzhou, China). The free amino acids present in the samples were calculated using the following formula: free amino acids (%) = c/6w × 100%, where c represents the mass concentration obtained from the linear regression equation based on the measured absorbance value, and w is the sample dry matter content. All analyses were performed with three biological replicates, each measured in duplicate.

### 2.3 Volatile metabolomics detection and analysis of tea leaves

The volatile compounds were extracted and analyzed using headspace solid phase microextraction/gas chromatography-mass spectrometry (HS-SPME/GC-MS) (Qiao et al., 2021). In summary, 2.0 g of tea and 0.0864 g of ethyl decanoate were added to a 40-mL headspace vial and promptly sealed. After preheating for 15 min at 80 °C, a divinylbenzene/carboxen/polydimethylsiloxane (DVB/CAR/PDMS) fiber (50/30 µm inner diameter, 2 cm length) (Supelco, Darmstadt, Germany) was inserted into the headspace vial, and extraction proceeded for 40 min at 80 °C. After extraction, the SPME fiber was injected into the GC inlet for 3 min at 250 °C.

GC-MS was carried out on an Agilent 7890B gas chromatograph coupled with a 5977A mass spectrometer (Agilent, Santa Clara, CA, USA). Compounds were separated on an HP-5MS column (30 m × 0.25 mm × 0.25 µm film thickness). The samples were injected in splitless mode. The carrier gas was high-purity helium (purity ≥ 99.99%), maintained at a column flow rate of 1.0 mL/min. The heating program had an initial temperature of 50 °C for 1 min, then increased to 220 °C for 5 min at the rate of 5 °C/min. The ion source temperature was 230 °C, and the electron impact (EI) ionization was 70 eV. The solvent delay time was measured to be four minutes. The scan range was 30–400 atomic mass units (amu). All analyses were performed with three biological replicates, each analyzed by GC-MS with a single injection per sample.

The qualitative identification of volatile components was achieved through the utilization of retention index (RI) and mass spectral match factors. RI was calculated by comparing retention times of a series of n-alkanes (C9–C21) to the values provided in the NIST 11 database that used the same capillary column as:


RI=100n+100[RT(x)−RT(n)]/[RT(n+1)−RT(n)]

RT(x) denotes the retention time of compound x. RT(n) and RT(n+1) represent the retention times of the alkanes with carbon number n and n + 1, respectively. These alkanes elute immediately before and after the compound x. The acceptance of compound matches was contingent upon the fulfillment of two criteria: first, the calculated RI must be less than 15; and second, the mass spectral match factor must exceed 90.

### 2.4 RNA sequencing and transcriptome analysis

The LD, YD, YL, and YY samples were utilized for the purpose of transcriptome sequencing, with three replicates being conducted for each sample. The samples were ground to powder using liquid nitrogen. The RNA extraction process was carried out in accordance with the protocol outlined in the kit’s instructions (hipure plant RNA mini kit B, R4151-02B, Magen Biotechnology Co., Ltd., Guangzhou, China). RNA concentration and integrity were assessed on 2% agarose gels and with a 2100 Bioanalyzer (Agilent, Santa Clara, CA, USA).

The quality control of transcriptome raw data was performed using fastp to filter out low-quality data (Chen et al., 2018). The short-read matching tool bowtie2 was utilized to align the clean reads against the ribosome database and to remove unmapped reads (Langmead and Salzberg, 2012). Global and local alignment searches were performed using HISAT2 to match spliced reads in RNA-seq data (Kim et al., 2015). The fragments per kilobase of exon model per million mapped fragments (FPKM) method was applied to calculate gene expression levels (Li and Dewey, 2011; Pertea et al., 2015). The differentially expressed genes (DEGs) were annotated with GO terms, and the number of genes associated with each GO function was determined. The hypergeometric test was applied to identify KEGG pathways that showed significant enrichment among the DEGs.

### 2.5 Statistical analysis

GraphPad Prism 10.0 was utilized for data visualization and statistical analysis. All data are presented as mean ± standard deviation (SD) calculated from three biological replicates. One-way analysis of variance (ANOVA) was performed to compare means among multiple groups, followed by Tukey’s honestly significant difference (HSD) *post hoc* test for multiple comparisons. Statistical significance was set at p < 0.05, and different lowercase letters above bars in figures indicate significant differences among groups based on the *post hoc* test results. For transcriptomic enrichment analyses (KEGG), p-values were adjusted for multiple testing using the Benjamini-Hochberg false discovery rate (FDR) correction, with FDR < 0.05 considered statistically significant. SIMCA 14.1 software was used for PLS-DA analysis and for the plotting of PLS-DA score plots and VIP value plots. Excel 2021 was used for the performance of Z-score standardization on gene expression data and for the plotting of heatmaps.

## 3 Results

### 3.1 Rootstock limits phenylpropanoid flux and reduces bitter taste components

To evaluate the regulatory effect of rootstock on the scion’s taste profile, we quantitatively analyzed the accumulation of catechins, caffeine, and free amino acids in the four groups (LD, YD, YL, YY).

**Figure 1:**
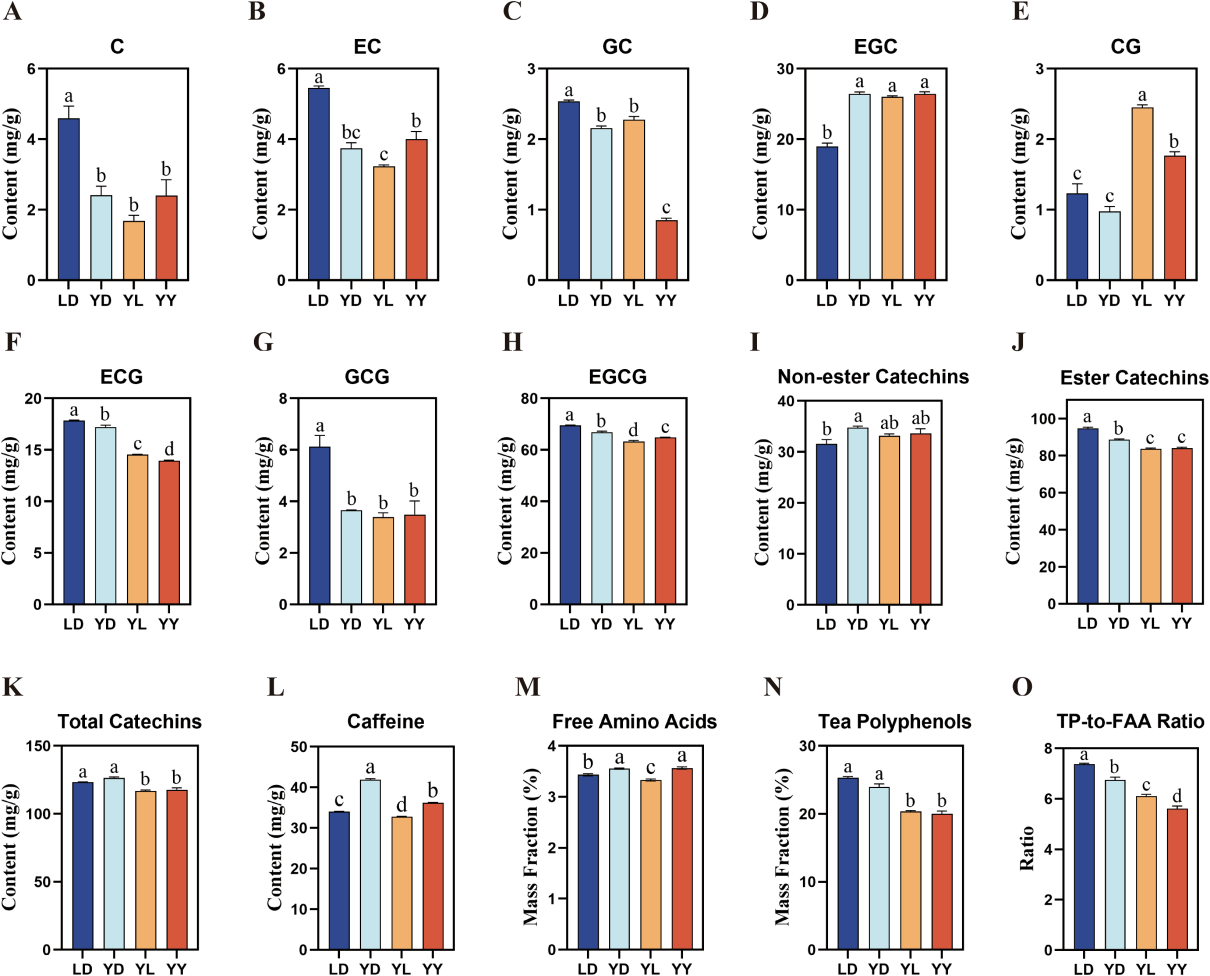
Profiles of non-volatile characteristic metabolite contents in four groups of tea leaves. The quantification of **(A–H)** catechins, **(I)** non-ester catechins**, (J)** ester catechins, **(K)** total catechins, **(L)** caffeine, **(M)** free amino acids (FAA), **(N)** tea polyphenols (TP), and **(O)** TP-to-FAA ratio in tea plants of LD, YD, YL, and YY. Different lowercase letters on top of bars indicate significant differences among the four groups at P < 0.05. LD, non-grafted ‘Lingtou Dancong’; YD, non-grafted ‘Yashixiang Dancong’; YL, hetero-grafting, ‘Yashixiang Dancong’ was scion and grafted onto ‘Lingtou Dancong’ rootstock; YY, homo-grafting, ‘Yashixiang Dancong’ was both used for scion and rootstock.

The data revealed a systematic suppression of the phenylpropanoid pathway in the hetero-grafted plants (**Figures 1A–H**). Specifically, while simple catechins (C, EGC, GCG) showed no significant variation among grafted groups (**Figures 1A, D, G**), the content of ester catechins (ECG and EGCG)—the primary contributors to bitterness and astringency—was significantly down-regulated (**Figures 1F, H**). Hetero-grafted ‘Lingtou’ (YL) plants exhibited significantly lower levels of ECG and EGCG compared to the non-grafted scion controls (YD). Notably, EGCG content in YL was significantly reduced even when compared to the homo-grafted YY group, suggesting that this suppression is driven specifically by the ‘Lingtou’ rootstock genotype rather than the grafting operation itself. Consistent with this trend, total catechin content in grafted plants was significantly lower than in non-grafted plants (**Figure 1K**). Caffeine content followed a similar repressive pattern: YL plants showed a significant reduction compared to both YD and YY (**Figure 1L**). Furthermore, the Tea Polyphenol-to-Free Amino Acid (TP/FAA) ratio—a key index of tea taste mellowness—was significantly lower in YL and YY compared to non-grafted controls (**Figure 1O**).

Collectively, these results indicate that the ‘Lingtou’ rootstock imposes a metabolic limitation on the synthesis of key taste components, suggesting a reallocation of carbon resources away from these non-volatile, defense-related phenylpropanoids and alkaloids. Importantly, the comparison between homo-grafting (YD-vs-YY) and hetero-grafting (YD-vs-YL) allowed us to dissect grafting-specific from rootstock genotype-specific effects. While homo-grafting induced modest metabolic changes (**Figures 1K, L**), the magnitude and direction of shifts in YL were substantially greater and qualitatively distinct, indicating that the ‘Lingtou’ rootstock genotype, rather than the grafting process itself, is the primary driver of the observed metabolic trade-off.

### 3.2 Rootstock-scion interaction promotes floral aroma compound accumulation

#### 3.2.1 Altered volatile profiles and enhanced aroma traits

Concurrently and in a compensatory manner, hetero-grafting triggered a significant enhancement of the volatile metabolome, counterbalancing the reduction in non-volatile compounds.

**Figure 2:**
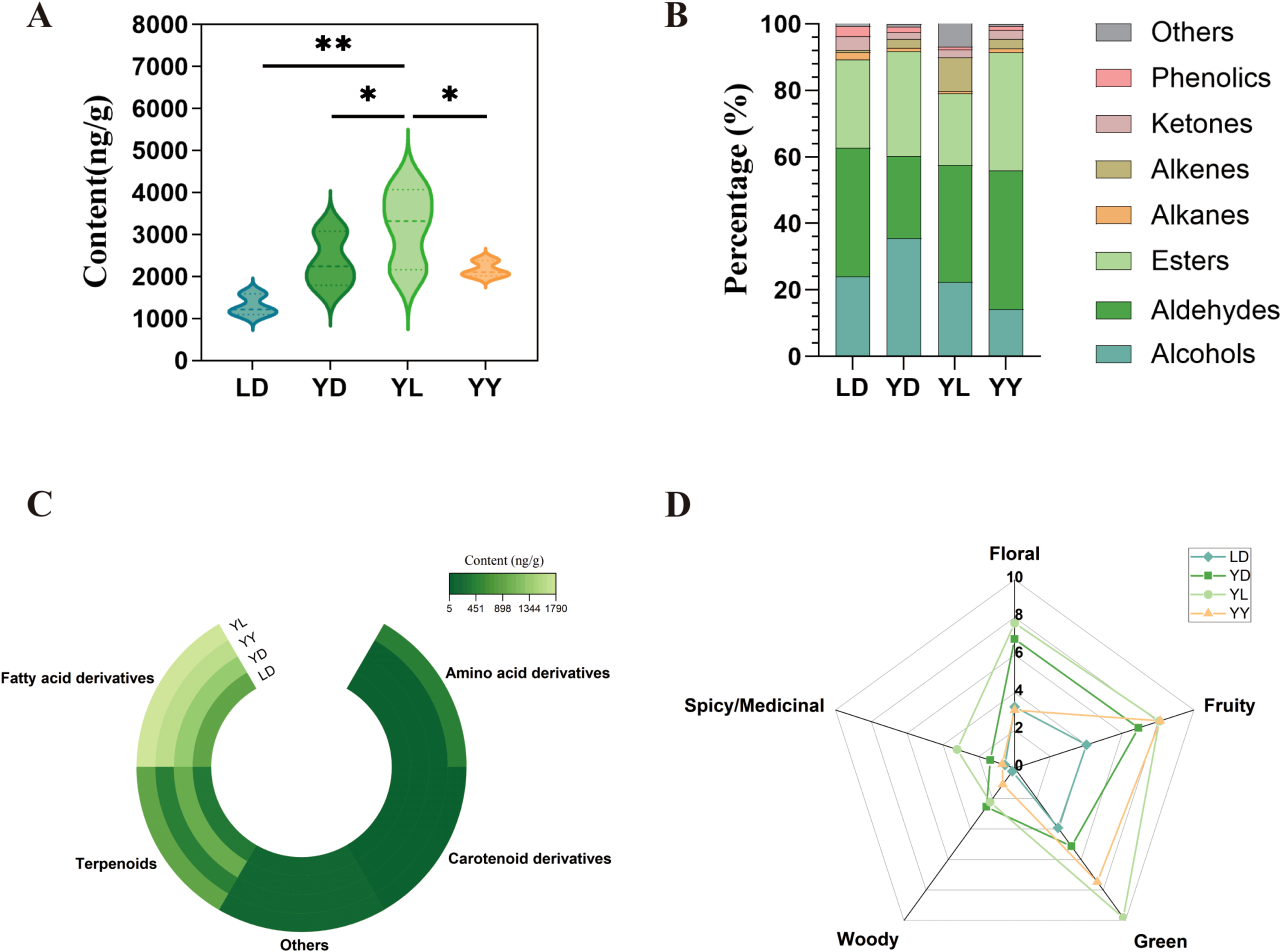
Overview of volatile metabolite content in four groups of tea leaves. **(A)** Total volatile metabolite content in tea leaves. **(B)** Percentage distribution of volatile metabolites classified by chemical structures. **(C)** Circular heatmap of volatile metabolite content composition categorized by synthetic precursor sources. **(D)** Radar plot of volatile metabolite content distribution across different aroma types.

A total of 68 volatile metabolites were identified. Quantitative analysis showed that the hetero-grafted YL group accumulated the highest total volatile content among all four groups (**Figure 2A**). Metabolic profiling revealed a structural reorientation of the aroma profile. Grafting induced a significant shift from alcohols to aldehydes and markedly promoted the synthesis of alkenes in the YL group (**Figure 2B**). When categorized by biosynthetic origin, YL plants exhibited the highest levels of fatty acid derivatives, terpenoids, and amino acid derivatives (**Figure 2C**). Sensory attribute mapping further indicated that YL possessed the most comprehensive aromatic profile, with superior scores in Floral, Fruity, and Green notes (**Figure 2D**).

#### 3.2.2 Key differential volatile metabolites identified

**Figure 3:**
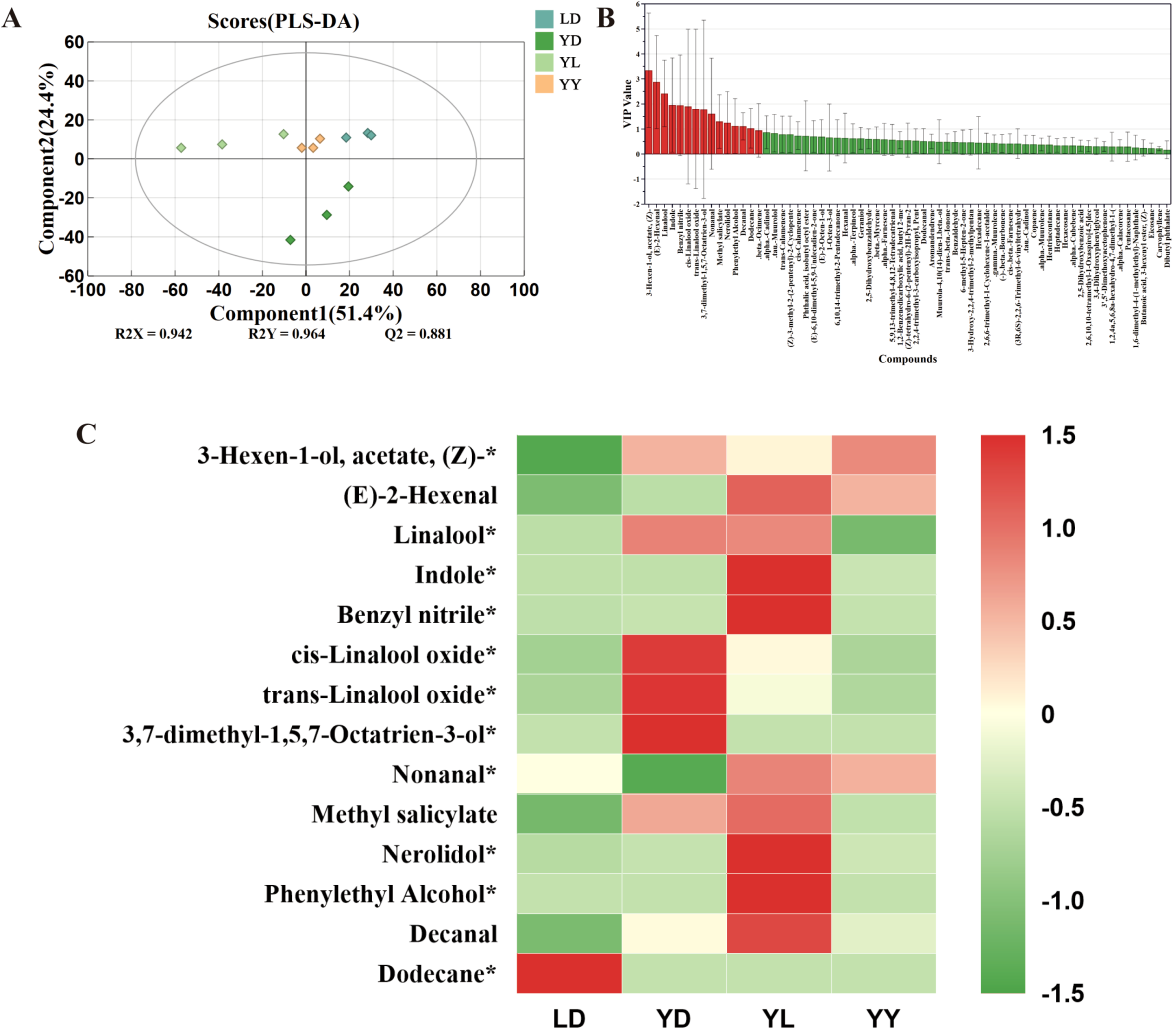
Screening of differential volatile metabolites and their content. **(A)** PLS-DA score plot of volatile metabolites in tea leaves. **(B)** VIP values of volatile metabolites in tea leaves. **(C)**Heat map of the content of screened differential volatile metabolites. (The asterisk (*) in the figure indicates significant differences in the volatile metabolites among the four groups of tea leaves.).

PLS-DA analysis (**Figure 3A**) confirmed distinct metabolic separation between groups. We identified 14 differentially expressed volatile metabolites (DEVMs) with VIP > 1 (**Figure 3B**). Crucially, 8 of these DEVMs were significantly enriched in the YL group, including key floral terpenoids like nerolidol and fatty acid-derived aldehydes such as (E)-2-hexenal, nonanal, and decanal (**Figure 3C**).

This specific accumulation pattern demonstrates that the rootstock actively redirects metabolic flux, favoring the synthesis of aromatic volatiles at the expense of taste-related phenylpropanoids, which is consistent with a rootstock-driven metabolic trade-off.

### 3.3 Transcriptional reprogramming underpins the metabolic trade-off

#### 3.3.1 Global transcriptomic changes and KEGG enrichment analysis of DEGs

**Figure 4:**
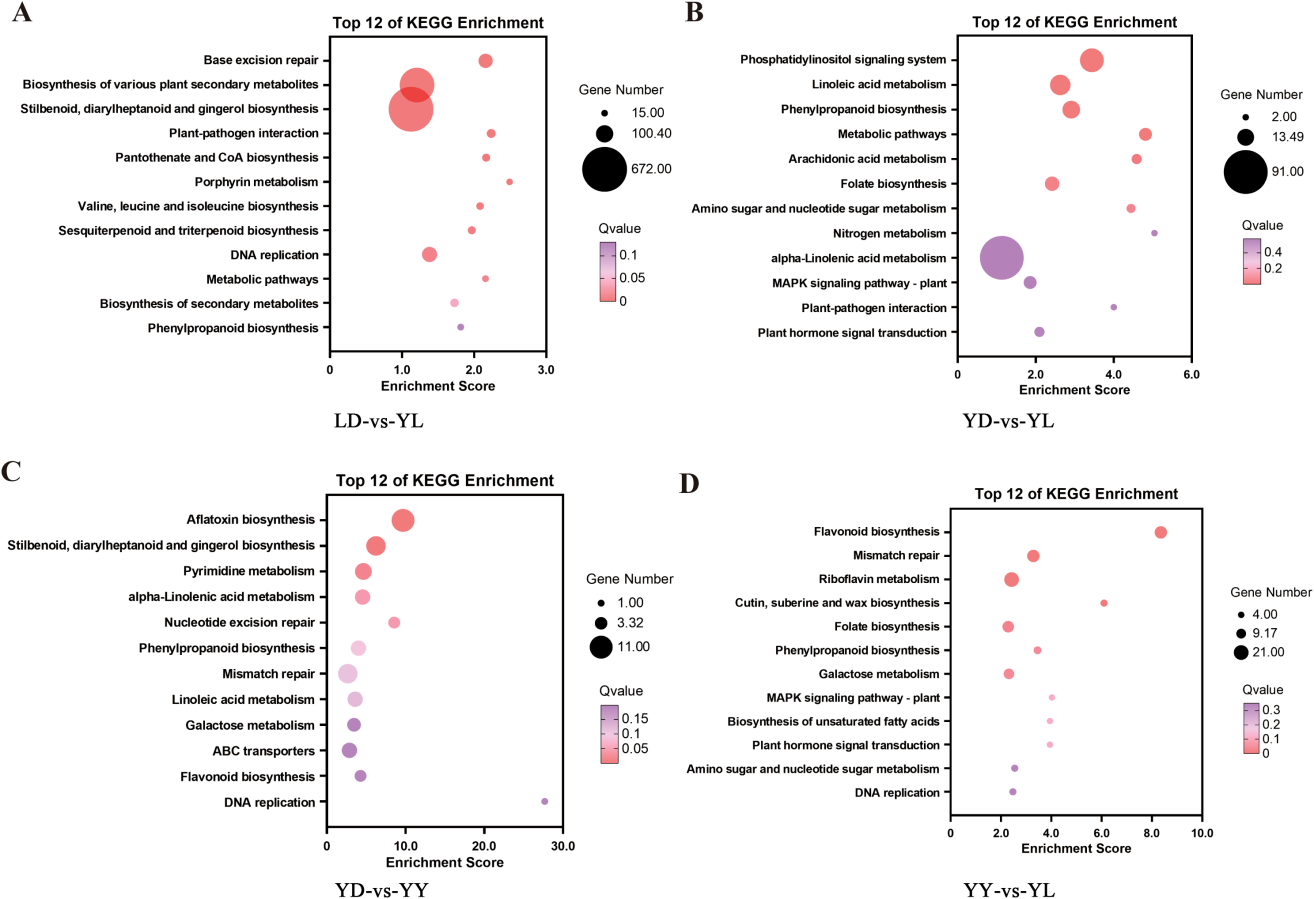
KEGG enrichment bubble heat map of DEGs in the four groups. DEGs distribution in the top 12 of KEGG Enrichment pathways of **(A)** LD-vs-YL, **(B)** YD-vs-YL, **(C)** YD-vs-YY, and **(D)** YY-vs-YL.

To elucidate the molecular drivers behind this “Taste-vs-Aroma” trade-off, we performed a comprehensive transcriptomic landscape analysis. A total of 9,817 DEGs were identified across the four comparison groups. Specifically, the YD-vs-YL (scion control vs. hetero-grafting) comparison revealed the most dramatic transcriptional response with 4,582 DEGs (2,145 upregulated and 2,437 downregulated), whereas the homo-grafting (YD-vs-YY) yielded only 1,243 DEGs (**Figure 4**). This about 3.7-fold difference in transcriptional response magnitude demonstrates that the rootstock’s genetic background, rather than the physical grafting wound or the scion genotype, is the dominant force reshaping the scion’s transcriptome.

#### 3.3.2 Expression patterns of key genes in flavor compound biosynthesis pathways

Transcriptomic profiling revealed a rootstock-induced, bidirectional transcriptional reprogramming that directly explains the metabolic trade-off. In the hetero-grafted YL group, a coordinated down-regulation created a “bottleneck” in the phenylpropanoid and caffeine pathways.

**Figure 5:**
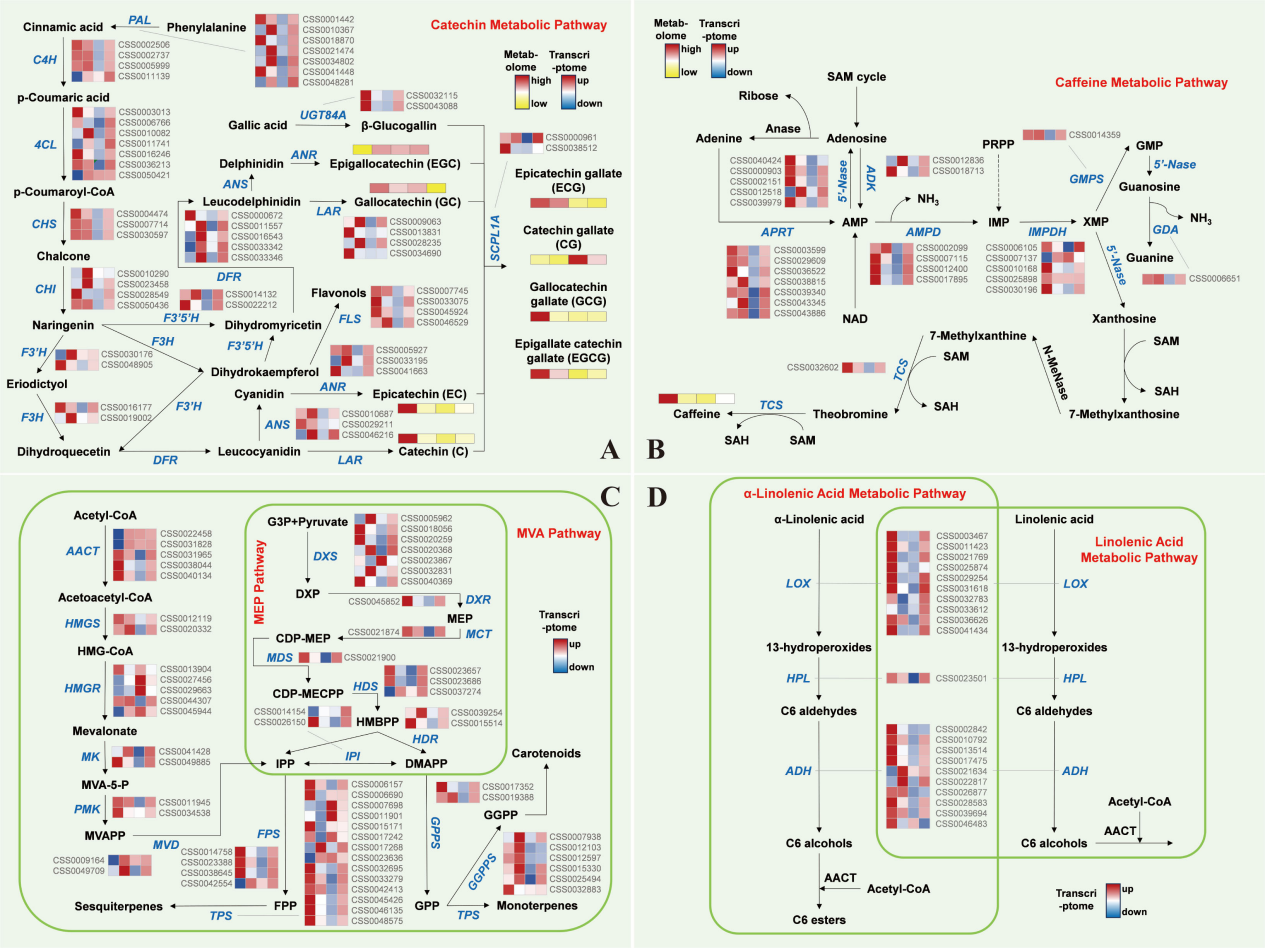
Metabolic pathways in tea plants and heat map of related gene expression. **(A)** Catechin metabolic pathway. **(B)** Caffeine metabolic pathway. **(C)** MVA pathway and MEP pathway. **(D)** α-Linolenic acid metabolic pathway and linolenic acid metabolic pathway. (The squares in the heat map are, from left to right, LD, YD, YL, and YY.).

Specifically, upstream flux control was evidenced by the significant suppression of key gateway genes, including *CsPAL* (Log_2_FC ranging from -1.8 to -2.3) and *Cs4CL*. Since PAL catalyzes the first committed step in the phenylpropanoid pathway, its downregulation effectively restricts carbon flow into all downstream flavonoids. Furthermore, genes specifically involved in the production of galloylated catechins, such as *CsF3’5’H* and *CsANS*, also showed a marked decrease in transcript abundance (**Figure 5A**). This molecular evidence directly explains the observed reduction in EGCG and ECG in the YL group. Regarding the caffeine pathway, the expression of *CsTCS* (the critical caffeine synthase) and *CsGMPS* was significantly lower in YL compared to YD and YY (**Figure 5B**), providing a clear transcriptional explanation for the reduced bitterness.

Simultaneously, and in a complementary fashion, hetero-grafting triggered a robust transcriptional activation of the aroma-related volatile pathways. We identified 42 DEGs enriched in the MVA and MEP pathways. The rate-limiting enzyme *CsHMGR* (Log_2_FC = 1.56) and *CsDXS* were significantly upregulated in YL (**Figure 5C**). This suggests that the ‘Lingtou’ rootstock enhances the supply of isopentenyl diphosphate (IPP), the universal precursor for all terpenoids. Downstream, multiple *CsTPS* (terpene synthase) genes, which catalyze the formation of floral volatiles such as nerolidol and β-ocimene, maintained high expression levels in YL. Additionally, the α-linolenic acid metabolism pathway, responsible for “green” aroma compounds, exhibited increased activity via the upregulation of *CsLOX* and *CsHPL* (**Figure 5D**).

Furthermore, we identified 108 differentially expressed transcription factors (TFs), primarily belonging to the MYB, WRKY, and bHLH families, which exhibited contrasting expression patterns between YD and YL (**Supplementary Tables 3**, **4**). The significant enrichment of MYB binding sites in the promoters of *CsPAL* and *CsTPS* suggests that the rootstock regulates the flavor trade-off by modulating these “master switches.” This dual-directional regulation—suppressing the phenylpropanoid “sink” while activating the terpenoid “source”—defines the unique molecular signature of hetero-grafted ‘Dancong’ tea.

### 3.4 Correlation networks identify key regulatory hubs

To identify the core regulatory hubs driving the observed metabolic trade-off, we integrated transcriptome and metabolome data through Weighted Gene Co-expression Network Analysis (WGCNA) and Pearson correlation networks (|*r*| > 0.80, *p* < 0.01) to identify the core regulatory hubs.

**Figure 6:**
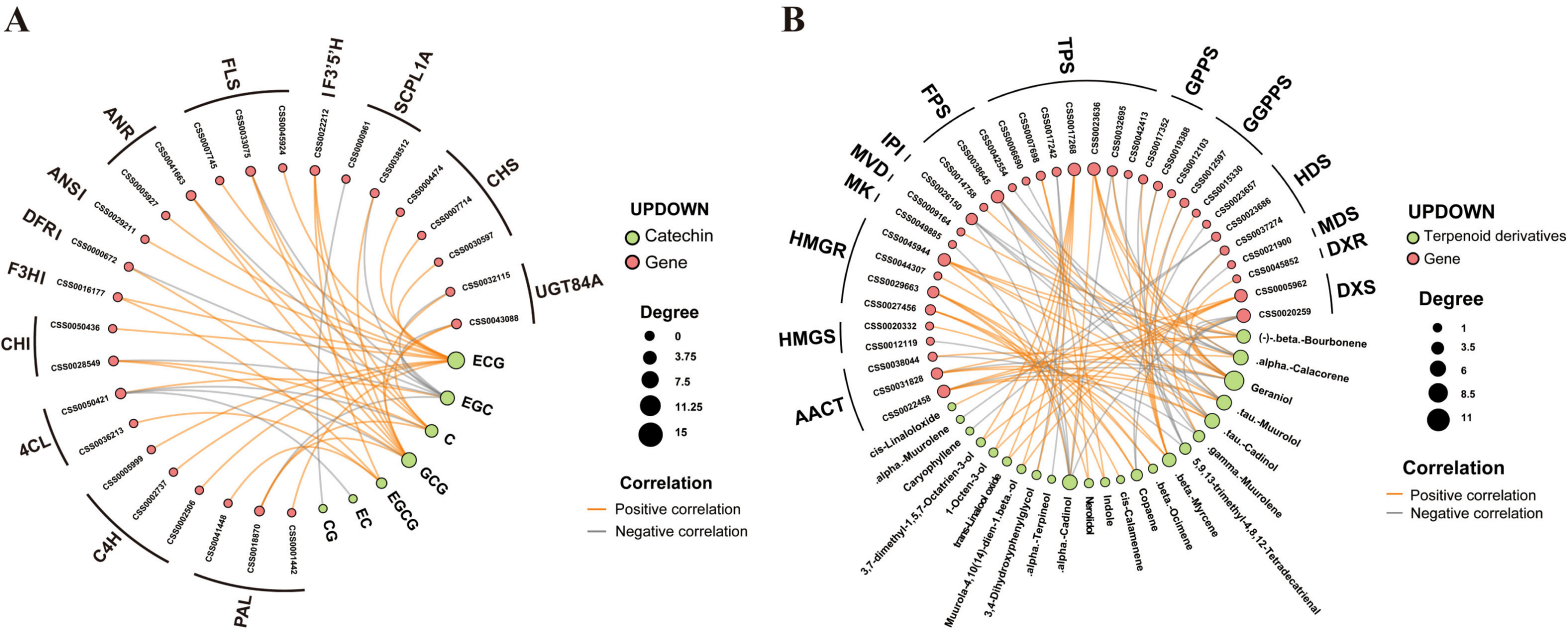
Metabolic pathway of terpenoids in tea plants and heat map of related gene expression. **(A)** Catechin-gene correlation web. **(B)** Terpenoids-gene correlation web.

In the catechin-gene regulatory network (**Figure 6A**), we identified a highly connected module where *CsPAL* and *Cs4CL* emerged as the central regulatory nodes. These hub genes exhibited strong topological connectivity with multiple non-volatile metabolites; specifically, *CsPAL* expression was positively correlated with the accumulation of EGCG (*r* = 0.92) and ECG (*r* = 0.88). The high degree of connectivity for *CsPAL* suggests that it serves as a primary control point for flux through the phenylpropanoid pathway in response to grafting. The synchronous repression of these hubs and their associated esterified catechins in the hetero-grafted YL group confirms their role as rate-limiting factors modulated by the rootstock-scion interaction.

In the aroma-gene regulatory network (**Figure 6B**), the complexity was significantly higher, reflecting the diverse chemical nature of the tea volatilome. *CsHMGR* and *CsTPS* were identified as the most influential hubs, with *CsTPS* showing strong positive correlations with key floral terpenoids such as nerolidol (*r* = 0.89), β-ocimene (*r* = 0.85), and linalool (*r* = 0.82). Notably, the network revealed that these terpenoid hubs are linked not only to backbone synthesis genes but also to specific MYB and WRKY transcription factors, which likely mediate the transmission of rootstock-derived signals to the aroma biosynthetic machinery. By integrating these multi-omics layers, we conclude that the flavor variation in grafted tea is a systemic reprogramming process controlled by a select group of hub genes—specifically *CsPAL* and *Cs4CL* for the reduction of astringency, and *CsHMGR* and *CsTPS* for the enhancement of aroma. These candidates provide high-priority targets for future molecular breeding aimed at optimizing tea quality by precision-tuning the balance between taste and fragrance.

## 4 Discussions

### 4.1 Rootstock-driven metabolic flux redirection: the trade-off between phenylpropanoids and terpenoids

Our integrated multi-omics analysis demonstrates that rootstock genotype does not merely “change” metabolism but induces a strategic reorientation of source-sink relationships. The significant reduction in phenylpropanoids (mainly ester catechins) and alkaloids (caffeine) coincided with the transcriptional bottlenecking of the phenylpropanoid pathway. Specifically, the strong negative correlation between the expression of gateway genes *CsPAL* and *Cs4CL* and the accumulation of phenylpropanoids, together with the known roles of these genes in the phenylpropanoid pathway, supports a model in which the ‘Lingtou’ rootstock restricts carbon flux into this metabolic sink. In parallel, the upregulation of *CsHMGR* (the rate-limiting enzyme of the MVA pathway) and *CsTPS* in hetero-grafted plants (YL) indicates a compensatory activation of the terpenoid backbone pathway. This inverse relationship supports a resource-driven metabolic trade-off: carbon skeletons and energy currency (ATP/NADPH) typically allocated to the high-cost phenylpropanoid “sink” (often associated with stress defense) are reallocated toward the synthesis of volatile terpenoids (associated with signaling and interaction). However, we acknowledge that these correlative data, while mechanistically consistent, do not constitute direct proof of causality. Comparisons with previous tea grafting studies reveal both conserved and genotype-specific responses. Qi et al. (2019) reported that grafting onto different rootstocks altered catechin profiles in scion leaves, consistent with our observation of reduced ester catechins in YL. Nevertheless, their study did not involve the transcriptome, while our transcriptomic data identify the specific molecular bottlenecks (*CsPAL*, *Cs4CL*) underlying this phenomenon. Similarly, Cheng et al. (2023) explored the changes in non-volatile metabolites and their molecular mechanisms but did not demonstrate the metabolic trade-off between phenylpropanoids and terpenoids. Our findings extend these earlier observations by revealing that the ‘Lingtou’ rootstock not only suppresses catechin accumulation but simultaneously redirects metabolic resources toward volatile terpenoids—a mechanistic insight not previously documented in tea.

The rootstock-driven metabolic trade-off observed here invites comparison with graft-induced metabolic regulation in other cropping systems. In herbaceous crops such as tomato and cucumber, grafting has been shown to modulate primary metabolites (sugars, organic acids) and volatile profiles, but the underlying mechanisms often involve source-sink relationships and hormone signaling rather than the phenylpropanoid-terpenoid antagonism documented here (Miao et al., 2019; Zhou et al., 2022). Interestingly, studies in woody perennials such as grapevine have reported rootstock-mediated shifts in secondary metabolism, including altered polyphenol profiles and volatile organic compounds, suggesting that woody plants may exhibit more pronounced trade-offs between defense and signaling metabolites due to their longer lifespan and greater investment in constitutive defenses (Cochetel et al., 2017). This distinction may reflect fundamental differences in metabolic resource allocation strategies between annual and perennial life histories. Our findings in tea, therefore, contribute to an emerging picture that woody perennials possess unique graft-responsive metabolic regulatory networks that merit further comparative investigation.

### 4.2 Reliability of candidate genes

While this study utilized correlation networks to identify key genes, the functional roles of the identified candidates are strongly supported by homology in model species. For example, the identified *CsTPS* and *CsHMGR* genes are well-established regulators of terpene accumulation in *Arabidopsis* and *Paeonia suffruticosa* (Wang et al., 2016; Niu et al., 2025). Additionally, correlation analysis indicated a strong positive link (Pearson > 0.80) between *CsTCS* expression and caffeine content, further supporting the regulatory role of the rootstock in modulating the purine alkaloid pathway.

Future functional validation, such as transient overexpression in tea leaves, virus-induced gene silencing (VIGS), or CRISPR-Cas9 editing, will be essential to establish direct causality and confirm the proposed bottleneck model. However, the current multi-omics dataset provides high-confidence candidates for such future breeding efforts.

### 4.3 Limitations and future directions

Although elucidating a clear metabolic mechanism, certain limitations warrant further investigation. Our model focused on a specific high-value combination (‘Yashixiang’/’Lingtou’) to ensure genetic homogeneity; expanding this to a broader panel of rootstock genotypes would help generalize the “rootstock-driven metabolic trade-off” model. Additionally, as graft compatibility and vascular connection mature over time, longitudinal studies are essential to determine the temporal stability of these transcriptional shifts. In perennial woody plants such as tea plants, grafting-induced metabolic changes may exhibit temporal dynamics due to the gradual establishment of fully functional vascular connections and the accumulation of systemic signals over multiple growing seasons (Goldschmidt, 2014; Warschefsky et al., 2016). For example, in grafted grapevine, rootstock-mediated effects on scion secondary metabolism have been shown to intensify over the first three years post-grafting as the root system matures (Cochetel et al., 2017). Therefore, while our one-year post-grafting dataset captures the initial reprogramming events, multi-year monitoring will be necessary to assess whether the observed phenylpropanoid-terpenoid trade-off is maintained, amplified, or attenuated as the plant enters full reproductive maturity. Future research should also focus on identifying the specific long-distance mobile signals (e.g., mRNA, small RNAs, or phytohormones) that travel from the rootstock to the scion to trigger this systemic reprogramming.

## 5 Conclusion

This study elucidates a rootstock-driven metabolic trade-off mechanism in *Camellia sinensis*, demonstrating that rootstock genotype actively reprograms the scion’s secondary metabolism. Specifically, the vigorous ‘Lingtou’ rootstock is associated with a transcriptional bottleneck the phenylpropanoid pathway (marked by downregulation of *CsPAL*) while simultaneously showing activation of the terpenoid backbone pathway (marked by upregulation of *CsHMGR*). This systemic regulation shifts the metabolic flux from defensive phenylpropanoids toward volatile terpenoids, thereby optimizing the balance between taste and aroma. These findings clarify the molecular mechanisms of flavor modulation in grafted tea plants, providing a physiological framework for using rootstock selection as a precision tool to modulate chemodiversity in grafted woody crops.

## Data Availability

The data presented in the study are deposited in the NCBI repository, accession number: PRJNA1446990.

## Supplementary material

Supplementary Figure 1 Non-grafted tea plants. **(A)** Non-grafted ‘Lingtou Dancong’ (LD). **(B)** Non-grafted ‘Yashixiang Dancong’ (YD).

Supplementary Figure 2 Schematic diagram of low-position cleft grafting technique for tea plants.

Supplementary Figure 3 Grafted tea plants. **(A)** YD scions grafted onto YD rootstock tea plants (YY, homo-grafting). **(B)** YD scions grafted onto LD rootstock tea plants (YL, hetero-grafting).
