# Supplementary figures and images for: Rootstock genotype drives a metabolic trade-off between phenylpropanoids and terpenoids in Camellia sinensis
Source: Front Plant Sci. 2026 Apr 16;17:1781298. doi: 10.3389/fpls.2026.1781298 (PMC13128568; doi:10.3389/fpls.2026.1781298)

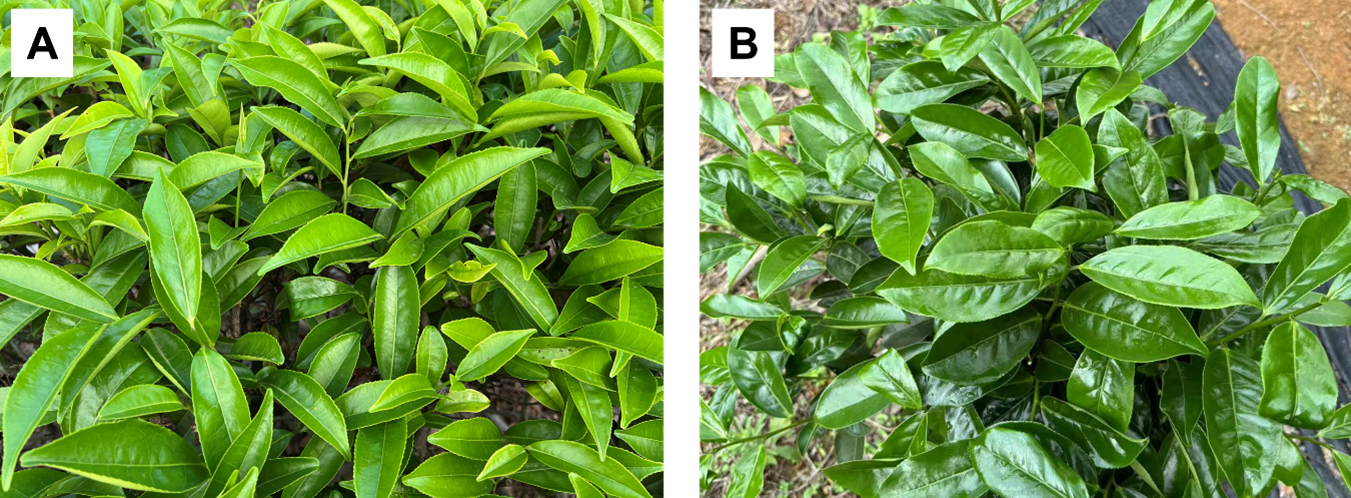

Supplement: Supplementary Figure 1 — Non-grafted tea plants. (A) Non-grafted ‘Lingtou Dancong’ (LD). (B) Non-grafted ‘Yashixiang Dancong’ (YD). [file Image1.tif]

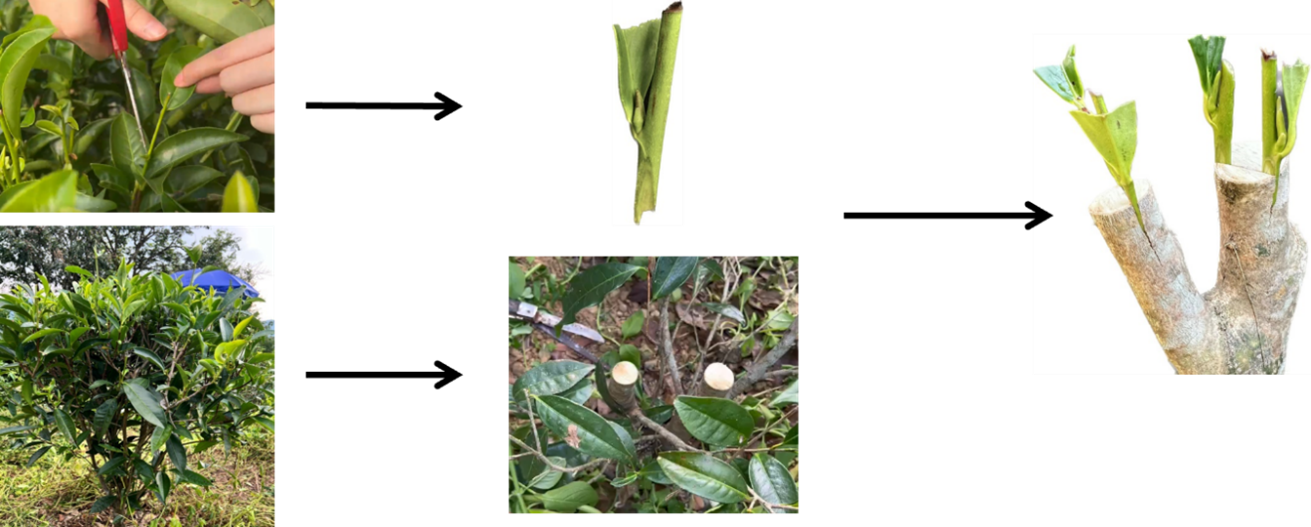

Supplement: Supplementary Figure 2 — Schematic diagram of low-position cleft grafting technique for tea plants. [file Image2.tif]

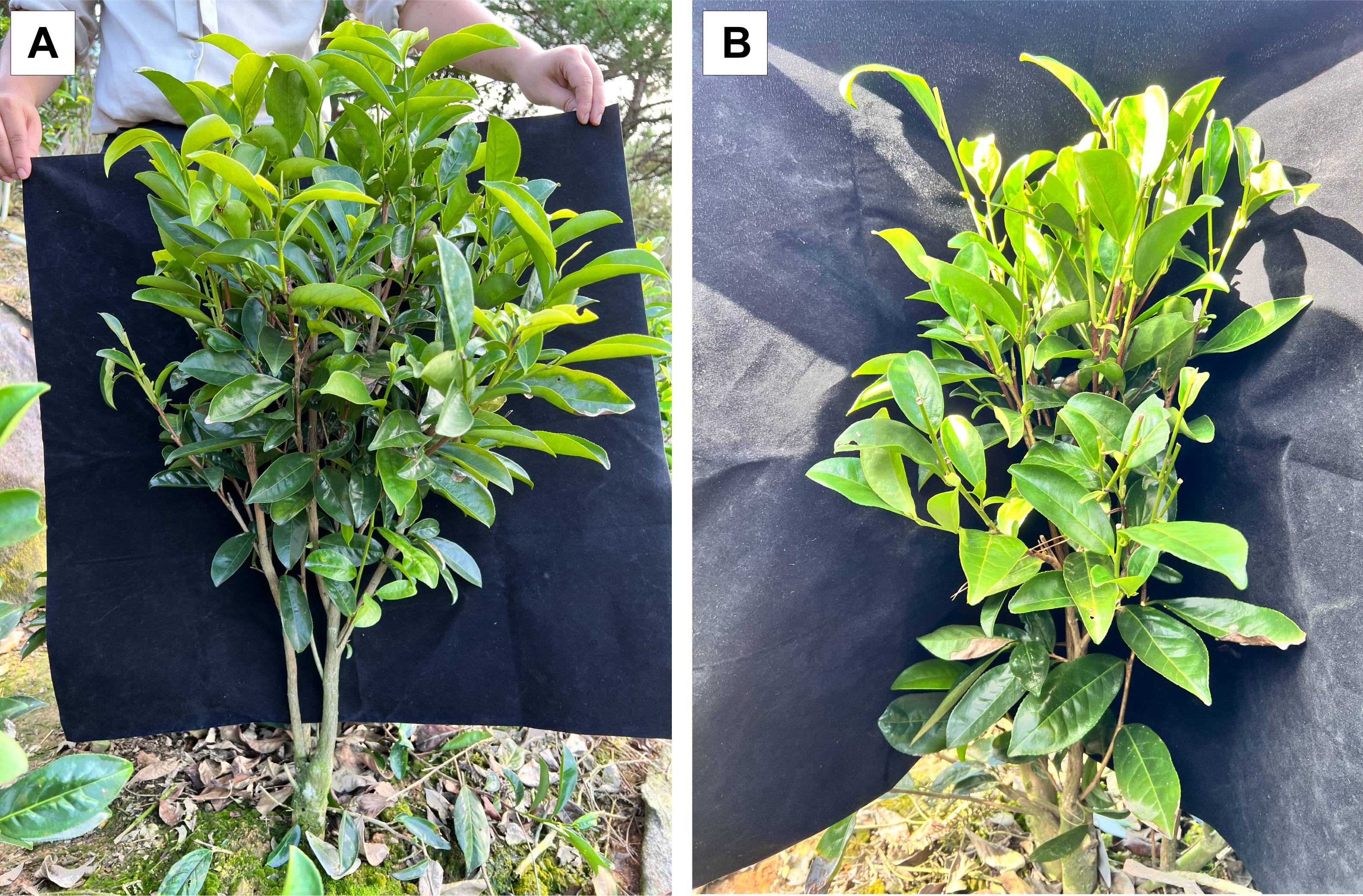

Supplement: Supplementary Figure 3 — Grafted tea plants. (A) YD scions grafted onto YD rootstock tea plants (YY, homo-grafting). (B) YD scions grafted onto LD rootstock tea plants (YL, hetero-grafting). [file Image3.tif]
